# Degree of foot process effacement in patients with genetic focal segmental glomerulosclerosis: a single-center analysis and review of the literature

**DOI:** 10.1038/s41598-021-91520-9

**Published:** 2021-06-08

**Authors:** Kiyonobu Ishizuka, Kenichiro Miura, Taeko Hashimoto, Naoto Kaneko, Yutaka Harita, Tomoo Yabuuchi, Masataka Hisano, Shuichiro Fujinaga, Tae Omori, Yutaka Yamaguchi, Motoshi Hattori

**Affiliations:** 1 Department of Pediatric Nephrology, School of Medicine, Tokyo Women’s Medical University, 8-1 Kawada-cho, Shinjuku-ku, Tokyo, 162-8666 Japan; 2 Department of Pediatrics, Yamagata University School of Medicine, Yamagata, Japan; 3 Department of Pediatrics, Graduate School of Medicine, The University of Tokyo, Tokyo, Japan; 4 Department of Nephrology, Chiba Children’s Hospital, Chiba, Japan; 5 Division of Nephrology, Saitama Children’s Medical Center, 1-2 Shintoshin, Chuo-ku, Saitama city, Saitama, 330-8777 Japan; 6 Department of Pediatrics, Tokyo Metropolitan Bokutoh Hospital, Tokyo, Japan; 7 Yamaguchi’s Pathology Laboratory, Chiba, Japan

**Keywords:** Kidney diseases, Genetics, Diseases, Nephrology

## Abstract

Determining the cause of focal segmental glomerulosclerosis (FSGS) has crucial implications for evaluating the risk of posttransplant recurrence. The degree of foot process effacement (FPE) on electron micrographs (EM) of native kidney biopsies can reportedly differentiate primary FSGS from secondary FSGS. However, no systematic evaluation of FPE in genetic FSGS has been performed. In this study, percentage of FPE and foot process width (FPW) in native kidney biopsies were analyzed in eight genetic FSGS patients and nine primary FSGS patients. All genetic FSGS patients showed segmental FPE up to 38% and FPW below 2000 nm, while all primary FSGS patients showed diffuse FPE above 88% and FPW above 3000 nm. We reviewed the literature which described the degree of FPE in genetic FSGS patients and identified 38 patients with a description of the degree of FPE. The degree of FPE in patients with mutations in the genes encoding proteins associated with slit diaphragm and cytoskeletal proteins was varied, while almost all patients with mutations in other FSGS genes showed segmental FPE. In conclusion, the present study suggests that the degree of FPE in native kidney biopsies may be useful for differentiating some genetic FSGS patients from primary FSGS patients.

## Introduction

Focal segmental glomerulosclerosis (FSGS) is one of the most frequent causes of end-stage kidney disease in children, and recurrence after kidney transplantation is a major challenge because of its association with poor graft survival^1^. FSGS is described as a renal histologic lesion with diverse causes and pathogenicity. Subclasses of FSGS include primary, genetic, and secondary forms, the latter of which comprises maladaptive, viral, and drug-induced FSGS^2–5^. Primary FSGS is caused by circulating factors and has a high risk of posttransplant recurrence, while other forms have very low risk of recurrence^1^. Therefore, identifying the cause of FSGS in each patient has crucial implications for the treatment strategy for kidney transplantation in these patients.

Advancements in next-generation sequencing techniques have allowed for rapid and efficient genetic variant detection. It has been proposed that genetic testing should be performed in all patients with child-onset steroid-resistant nephrotic syndrome^6^. However, genetic testing may not be feasible in some situations, especially when insurance coverage is not available for the test^7^. In addition, a negative test result does not exclude genetic disease, as novel mutations in undiscovered genes may be missed^3^. Therefore, thorough clinicopathologic evaluations remain an indispensable measure to identify the cause of FSGS.

Deegens et al. analyzed the differences in foot process width (FPW) between patients with primary FSGS versus those with secondary FSGS and found the effacement to be most severe in those with primary FSGS. Foot process was relatively preserved in secondary FSGS, with little overlap between the two subclasses^8^. Sethi et al. described that FSGS patients with nephrotic syndrome showed diffuse foot process effacement (FPE) in electron microscopy (EM) images, whereas those without nephrotic syndrome showed segmental FPE. The authors concluded that EM findings in native kidney biopsies are useful for differentiating primary FSGS from secondary FSGS^9^.

However, to date, no systematic evaluation of FPE in genetic FSGS has been performed. In this study, we analyzed the degree of FPE by EM analysis of native kidney biopsies in a case series with genetic FSGS and also reviewed the literature describing the degree of FPE in genetic FSGS. Additionally, we examined the degree of FPE in patients with a definitive diagnosis of primary FSGS who had a posttransplant recurrence. Finally, we examined whether the degree of FPE seen in EM images can differentiate genetic FSGS from primary FSGS.

## Results

### Baseline demographics and clinical data

There were no significant differences observed between primary FSGS and genetic FSGS with respect to age at disease onset, sex, time from onset to end-stage kidney disease, urinary protein excretion at kidney biopsy, and the Columbia classification (Table 1). The proportion of patients with edema was significantly higher in patients with primary FSGS than in those with genetic FSGS. Notably, five of eight patients with genetic FSGS met the criteria of nephrotic syndrome at kidney biopsy, and two of the five who met the criteria showed systemic edema during the clinical course. No patients with maladaptive FSGS presented with nephrotic syndrome or systemic edema.

**Table 1:** Demographics and clinical data of patients.

	Primary FSGS	Genetic FSGS	Maladaptive FSGS
n	9	8	3
Age at onset (yrs)	4.2 [3.4, 7.3]	3.3 [2.9, 5.4]	5.4 [4.2, 7.1]
Sex (male/female)	7/2	4/4	2/1
Time from onset to ESKD (yrs)	6.5 [1.9, 7.9]	4 [1.9, 6.7]	
Urinary protein to creatinine ratio (g/g) at kidney biopsy	9.8 [8.3, 10.5]	3.2 [1.7, 6.2]	1.0 [0.6, 1.4]
Serum TP level (g/dl) at kidney biopsy^**a**^	3.5 [3.4, 4.5]^b^	5.6 [4.7, 6.3]^b^	6.1 [6.0, 6.3]
Nephrotic syndrome (yes/no)	9/0	5/3	0/3
Systemic edema during clinical course (yes/no)	9/0^c^	2/6 ^c^	0/3
**Columbia classification of FSGS**			
Collapsing	5	3	0
Tip lesion	0	0	0
Cellular	1	0	0
Perihilar	0	3	2
Not otherwise specified	3	2	1

ESKD, end-stage kidney disease; FSGS, focal segmental glomerulosclerosis; TP, total protein.

Data was expressed as medians with 25th and 75th percentiles.

^a^Serum total protein level, instead of serum albumin level, was used for the definition of nephrotic syndrome, because some patients in this study were lacking in records of serum albumin levels at native kidney biopsies.

^b^*p* = 0.0125, ^c^*p* = 0.003.

### Genetic mutations

Pathogenic mutations identified in patients with genetic FSGS (patient numbers 1–8) are shown in Supplementary Table 1. The affected genes were *NUP107* in three patients^10^, *WT1* in two patients, and *LAMB2*, *INF2*, and *NUP93*^11^ in one patient each. No patients with primary FSGS had pathogenic mutations in the 64 genes analyzed in the present study.

### The degree of FPE in each group

**Figure 1:**
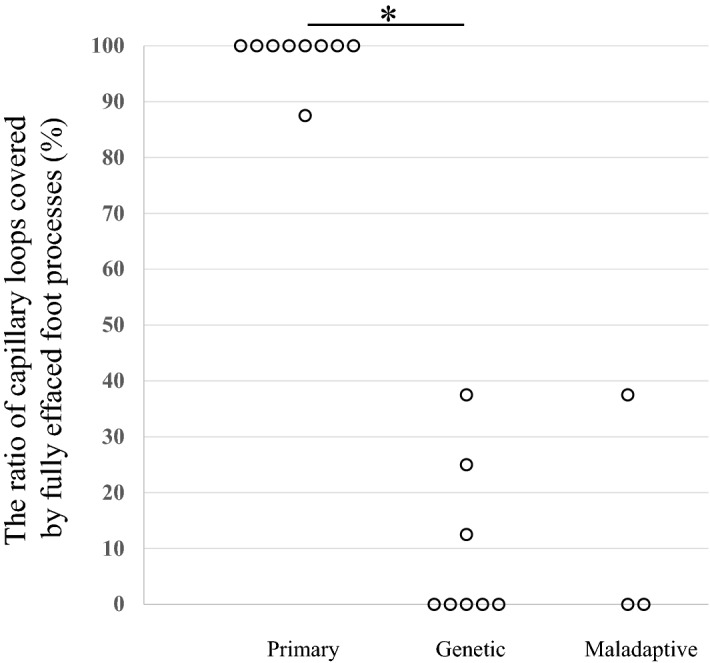
The degree of FPE (%FPE), shown as the percentage of capillary wall surface that was covered by podocyte foot processes uninterrupted by filtration slits. All patients with genetic FSGS (eight patients) showed segmental FPE ranging from 0 to 38%, while all patients with primary FSGS (nine patients) showed diffuse FPE ranging from 88 to 100%. Percentage of FPE was significantly higher in primary FSGS patients than in genetic FSGS patients (*p* = 0.0003). Percentage of FPE of maladaptive FSGS (three patients) ranged from 0 to 38%.

Percentage of FPE in primary, genetic and maladaptive FSGS patients is shown in Fig. 1. Percentage of FPE in genetic FSGS patients ranged from 0 to 38%, while that in primary FSGS patients ranged from 88 to 100%. Therefore, all patients with genetic FSGS showed segmental FPE and all patients with primary FSGS showed diffuse FPE (Fig. 1). Percentage of FPE was significantly higher in primary FSGS patients than in genetic FSGS patients (*p* = 0.0003). Percentage of FPE in maladaptive FSGS patients ranged from 0 to 38%.

**Figure 2:**
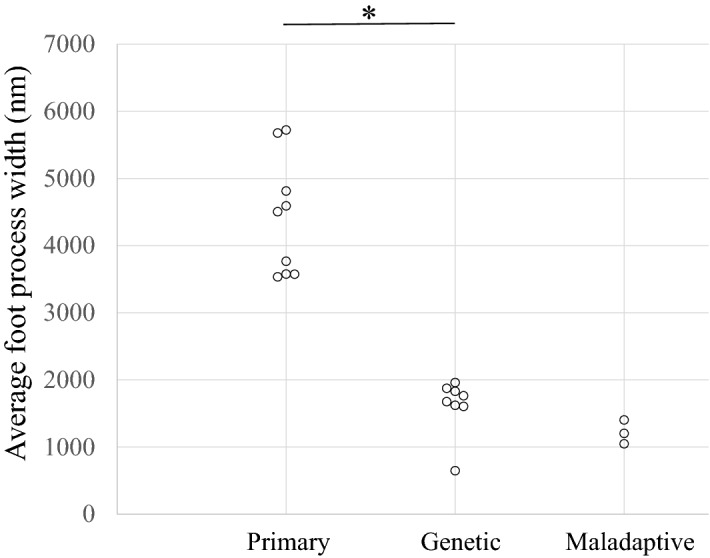
Foot process width of patients with primary, genetic and maladaptive FSGS patients. Median FPW was 4504 nm (range, 3534–5722 nm), 1719 nm (range, 647–1960 nm), and 1203 nm (range, 1047–1402 nm) in primary, genetic, and maladaptive FSGS patients, respectively. FPW was significantly larger in primary FSGS patients than in genetic FSGS patients (*p* = 0.0006).

**Figure 3:**
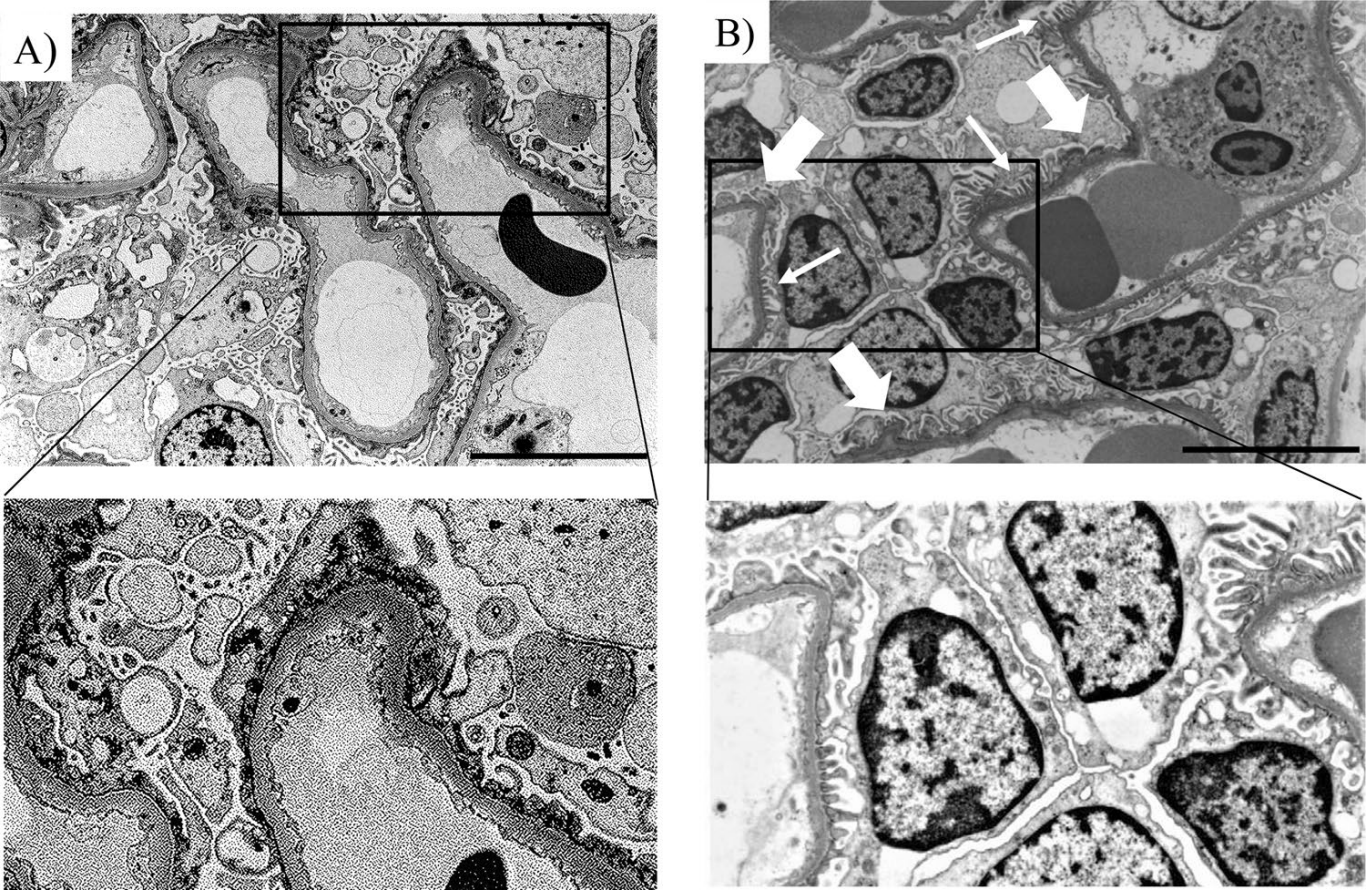
Representative electron micrographs of a patient with (**A**) primary FSGS and one with (**B**) genetic FSGS. (**A**) The patient (No. 13) with primary FSGS showed 100% FPE. All capillary loops were fully covered by FPE. (**B**) The patient (No. 1) with genetic FSGS (*NUP107* mutation) showed segmental (0%) FPE with no capillary loops fully covered by FPE. The thin white arrows indicate preserved foot processes, and the thick white arrows point to effaced foot processes. Lower panels show images with a higher magnification. Original magnification: 3000× in (**A**) and (**B**). The scale bar denotes 10 μm.

FPW in primary, genetic, and maladaptive FSGS patients is shown in Fig. 2. FPW of all genetic FSGS patients was below 2000 nm, while that of all primary FSGS patients was above 3000 nm. FPW of all maladaptive FSGS patients was below 1500 nm. FPW was significantly larger in primary FSGS than in genetic FSGS (*p* = 0.0006) (Fig. 2). Representative electron micrographs in a patient with primary FSGS and a patient with genetic FSGS are shown in Fig. 3A,B, respectively.

### The relationships between the amount of proteinuria and the degree of FPE (Supplementary Fig. 1)

Because less patients with genetic FSGS patients showed nephrotic syndrome compared to primary FSGS patients (Table 1), we examined the relationships between the amount of proteinuria and the degree of FPE. The amount of proteinuria correlated with neither percentage of FPE (*r* = 0.44; *p* = NS) nor FPW (*r* = 0.39; *p* = NS).

### Literature review of articles and case reports describing the degree of FPE in genetic FSGS patients

A total of 1768 articles were identified using the predefined search strategy. By screening the study titles and abstracts, 1111 were considered not eligible as they did not address EM findings of patients with pathogenic mutations in the genes analyzed in this study. Subsequently, 640 of the remaining 657 studies were excluded after full review for the following reasons: 88 articles described patients with congenital or infantile nephrotic syndrome; 552 articles did not provide description of the FPE. Together with eight articles found by manual search, a total of 25 articles consisting of one review article, two case series, and 22 case reports describing a total of 38 cases were included^12–36^. Mutated genes identified in these 38 patients included *CD2AP*, *KIRREL1*, *TRPC6*, *ACTN4*, *INF2*, *CRB2*, *PLCE1*, *WT1*, *NUP93*, *LAMB2*, *ITGA3*, and *COL4A3*. Patients with *NPHS1* mutations were excluded because the disease onset was in infancy in all patients. Our study included three patients who were described in the previous reports^10,11^ and five patients who were not described previously. The degree of FPE in a total of 46 patients from the literature and the present study is summarized in Table 2. Patients with pathogenic mutations in the genes that encode proteins associated with slit diaphragm, such as *NPHS2*^12–14^, *CD2AP*^15^, *KIRREL1*^16^, and *TRPC6*^17–19^ showed diffuse FPE, except for one case with *NPHS2* mutations^13^. Patients with mutations in the genes that encode cytoskeletal proteins, such as *ACTN4*^20–23^ and *INF2*^24–26^, showed varied degrees of FPE, with some patients showing segmental FPE and others showing diffuse FPE. All patients with mutations in the genes that encode other functioning proteins associated with podocytes and glomerular basement membrane (GBM) showed segmental FPE, except for one case with a *WT1* mutation^31^.

**Table 2:** Foot process effacement in genetic FSGS, as demonstrated in published literature and the present study.

Gene	Protein	Degree of FPE described^a^	References
**Slit diaphragm associated proteins**			
*NPHS2*	Podocin	Extensive (2 cases)	^12^
		Diffuse (2 cases)	^13^
		Segmental	^13^
		Extensive (2 cases)	^14^
*CD2AP*	CD2-associated protein	Widespread	^15^
*KIRREL1*	kin of IRRE-like protein 1	Extensive	^16^
*TRPC6*	Transient receptor potential cation channel, subfamily c, member 6	Diffuse	^17^
		Diffuse	^18^
		Diffuse	^19^
**Cytoskeletal proteins**			
*ACTN4*	α-actinin 4	Preserved	^20^
		Extensive	^21^
		Segmental	^21^
		Segmental (4 cases)	^22^
		Diffuse	^23^
*INF2*	Inverted formin 2	Focal	^24^
		Segmental (2 cases)	^25^
		Extensive	^26^
		Diffuse	^26^
		Segmental	this study^b^
**Apical proteins**			
*CRB2*	Crumbs family member 2	Less extensive	^27^
		In a small area	^28^
		Segmental	^29^
**Cell signaling associated proteins**			
*PLCE1*	Phospholipase C epsilon 1	Minimal	^13^
		Well preserved	^3^
**Nuclear protein and transcriptions factors**			
*WT1*	Wilms’ tumour protein 1	Segmental (2 cases)	This study^b^
		Segmental	^30^
		Extensive	^31^
		Segmental	^32^
*NUP93*	Nuclear pore complex protein 93	Partial (2 cases)	^33^
		Segmental	This study^b^^11^
*NUP107*	Nuclear pore complex protein 107	Segmental (2 cases)	This study^b^^10^
		Segmental	This study^b^
**Glomerular basement membrane-associated proteins**			
*LAMB2*	Laminin subunit β	Segmental	^34^
		Segmental	This study^b^
*ITGA3*	Integrin alpha-3	Partial/abnormal	^35^
*COL4A3*	Type IV collagen alpha 3	Localized	^36^

FPE, foot process effacement.

^a^The degree of FPE was shown according to the description in each literature.

^b^Patients included in this study.

## Discussion

This study is the first to examine the degree of FPE in a case series of genetic FSGS patients and compare them with those in children with a definitive diagnosis of primary FSGS who had posttransplant recurrence. Children with maladaptive FSGS were also analyzed and showed segmental FPE (Fig. 1), which was consistent with a previous report^9^. Furthermore, FPW in all maladaptive FSGS patients was lower than 1500 nm, which was also consistent with the description by Deegens et al.^8^. All patients with genetic FSGS included in this study showed segmental FPE (%FPE < 40%), while all patients with primary FSGS showed diffuse FPE (%FPE > 80%) (Fig. 1). Additionally, FPW of all genetic FSGS patients was below 2000 nm, while that of all primary FSGS patients was above 3000 nm (Fig. 2). Therefore, our results suggest that the degree of FPE seen in EM images may be helpful to discriminate between some genetic FSGS patients and primary FSGS patients.

Several studies showed that the degree of FPE correlated with the amount of proteinuria^37,38^. Sethi et al. reported that FSGS patients presenting with nephrotic syndrome and diffuse FPE in EM images are likely to have primary FSGS^9^. In the present study, the amount of proteinuria correlated with neither percentage of FPE nor FPW (Supplementary Fig. 1). Additionally, urine protein excretion was not significantly different between patients with primary FSGS and those with genetic FSGS (Table 1). Notably, some patients with genetic FSGS presented with nephrotic syndrome and/or systemic edema, suggesting that these clinical manifestations are less helpful to discriminate between primary FSGS and genetic FSGS. Therefore, our study suggested that the degree of FPE seen in EM images may contribute to identifying primary FSGS and some cases of genetic FSGS, regardless of the presence or absence of nephrotic syndrome.

Our literature review identified 38 patients with genetic FSGS whose EM images were analyzed for the degree of FPE. As shown in Table 2, previous case reports and our results suggest that patients with mutations in the genes encoding slit diaphragm-associated proteins showed diffuse FPE, whereas those with mutations in the genes that encodes cytoskeletal scaffold and membrane proteins showed varied degrees of FPE. Almost all patients with mutations in the genes that encodes other proteins associated with podocytes and the GBM showed segmental FPE. The functions and localization of affected podocyte genes may impact the degree of FPE in genetic FSGS patients. For *NPHS2*, truncating or homozygous R138Q mutations resulted in earlier onset of disease before six years of age, while it was significantly later in patients with any other *NPHS2* mutation, indicating a genotype–phenotype correlation^39^. Additionally, two siblings have been reported to have different clinical features with the degree of FPE: one showed diffuse FPE, while the other showed segmental, despite having the same genotype of *NPHS2* mutations^13^. Similar findings were reported in siblings who had *ACTN4* mutations^21^. These studies highlight a complex relationship between genotype, environmental factors, and epigenetic phenomena that is responsible for significant variability in the phenotype of a gene mutation. Combined with the results obtained from our patients, segmental FPE seen in EM images is strongly suggestive of genetic FSGS rather than primary FSGS.

This study is limited by a small sample size obtained from a single medical center as well as the diversity of genetic FSGS. The mutated genes identified in this study were different from the genes previously reported from Western countries. These studies described that the most frequently affected genes were *NPHS2* and *WT1* in patients with FSGS or steroid-resistant nephrotic syndrome at age of onset ≥ one year^40,41^. It has been reported that mutations in the *NPHS2* genes are rarely identified in Japanese children with FSGS^42,43^. Additionally, our study did not examine adult FSGS patients. Further studies in a larger number of patients with mutations in different genes are needed to fully investigate the degree of FPE in genetic FSGS patients.

In conclusion, our study suggests that the degree of FPE in native kidney biopsies may be useful for differentiating some genetic FSGS cases from primary FSGS cases, which will help with the evaluation of the risk of recurrence before kidney transplantation.

## Patients and methods

### Study population (Fig. 4)

**Figure 4:**
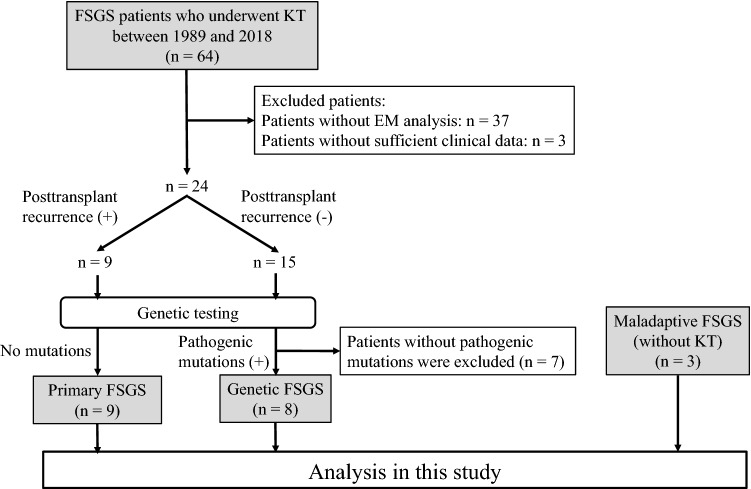
Study population in the present study. FSGS, focal segmental glomerulosclerosis; KT, kidney transplantation; EM, electron microscopy.

In this study, patients with congenital or infantile nephrotic syndrome were excluded, because they greatly differ in clinical manifestations and genetic background from FSGS patients with later onset^40,41,44^. A total of 64 patients with FSGS who underwent kidney transplantation at our institution between January 1, 1989 and December 31, 2018 were identified. No organs were procured from prisoners. All transplantations were performed at Tokyo Women’s Medical University. Thirty-seven kidney transplant recipients who were not analyzed by EM in their native kidney biopsies and three patients without sufficient clinical data to determine the presence of nephrotic syndrome were also excluded from this study. Of the remaining 24 patients, 9 showed posttransplant recurrence, while 15 did not. Clinical characteristics of the patients who showed posttransplant recurrence (patient numbers 9 to 17), and thus were diagnosed as having primary FSGS, are shown in Supplementary Table 2.

All 24 patients underwent genetic testing. We performed whole-exome sequencing using peripheral blood mononuclear cells with a focus on 64 genes currently known to be associated with FSGS (Supplementary Table 3). Of the 15 patients without posttransplant recurrence of FSGS, eight had pathogenic mutations in the genes associated with FSGS. Clinical characteristics of these eight patients with genetic FSGS (patient numbers 1 to 8) are shown in Supplementary Table 4. The remaining seven patients did not have any pathogenic mutations in the genes associated with FSGS. Because they did not experience posttransplant recurrence and may have as yet undiscovered genetic mutations associated with FSGS, a definitive diagnosis of primary FSGS could not be made, and thus were excluded from this study. Consequently, nine patients with a definitive diagnosis of primary FSGS and eight patients with genetic FSGS were included in this study. Three patients with maladaptive FSGS, diagnosed based on their clinical manifestations and native kidney biopsies between 1989 and 2018, were also included in this study to determine whether they show segmental FPE as previously reported^9^. The causes of maladaptive FSGS in these patients were bilateral hypoplastic kidneys, cyanotic congenital heart disease, and obesity-related nephropathy in one patient each. All three patients with maladaptive FSGS neither progressed to end-stage kidney disease nor underwent kidney transplantation. This study was approved by the ethical committees of Tokyo Women’s Medical University (approval number #4866-R3). All procedures performed in studies were in accordance with the 1964 Helsinki Declaration and its later amendments or comparable ethical standards. Informed consent was obtained from all individuals participating in this study.

### Definitions

Nephrotic syndrome has been defined as the presence of a urinary protein to creatinine ratio above 2.0 g/g^45^ and a serum total protein level ≤ 6.0 g/dl^46^. The serum total protein level, instead of the serum albumin level, was used to define nephrotic syndrome, because some patients in this study lacked records of serum albumin levels at native kidney biopsies. End-stage kidney disease was diagnosed when a patient required chronic dialysis or kidney transplantation. A diagnosis of posttransplant recurrence of FSGS was based on the presence of at least one of the following criteria: (1) clinical recurrence of the nephrotic syndrome; (2) graft biopsy showing diffuse FPE by EM; (3) histological identification of FSGS by light microscopy in the absence of transplant glomerulopathy or any other apparent cause of proteinuria^47^.

### Kidney pathology evaluation

Pathological findings of native kidney biopsies, including EM images, were analyzed in all patients. Light microscopy evaluation of kidney biopsies included staining with hematoxylin and eosin, periodic acid-Schiff, Masson’s trichrome, and periodic acid-methenamine-silver stain. Toluidine blue stained semi-thin sections were examined, and non-segmentally sclerosed glomeruli were identified for EM studies. Each biopsy was classified according to the Columbia classification^48^.

### Degree of FPE in EM images

We examined the degree of FPE using two methods, which was described by Sethi et al. and Deegens et al.^8,9^. Percentage of FPE was defined as the percentage of capillary wall surface that was covered by podocyte foot processes uninterrupted by filtration slits^9^. In brief, eight capillary loops within one glomerulus that was neither globally sclerosed nor collapsed were analyzed by EM at a magnification of 1000× to 3000× for each patient. If foot processes were preserved or only partially effaced in one loop, this loop was not judged as diffuse effacement. Percentage of FPE was defined as the percentage of the eight loops that showed complete effacement: 100%, all loops showed complete effacement; 88%, one of eight loops did not show complete effacement; 75%, two of eight loops did not show complete effacement. No more than eight capillary loops on electron micrographs were eligible for analysis because of the retrospective nature of this study, although the previous study used 10 loops in each patient^9^.

Average FPW was calculated by dividing the total number of foot processes by the total length of the GBM^8^. Eight capillary loops within one glomerulus that was neither globally sclerosed nor collapsed were analyzed by EM at a magnification of 1000× to 3000× for each patient. ImageJ software (National Institutes of Health, USA) was used to measure the length of the GBM for each loop. Also, for each loop the number of foot processes was manually counted.

### Immunosuppression regimen through kidney transplantation

Five patients (four of nine patients with primary FSGS and one of eight patients with genetic FSGS), who underwent kidney transplantation between April 1983 and January 2001, were treated with immunosuppressive regimens consisting of calcineurin inhibitor (cyclosporine or tacrolimus), azathioprine or mizoribine, and methylprednisolone^49^. Antilymphocyte globulin or deoxyspergualin was used as an induction agent. In the remaining 12 patients who underwent kidney transplantation between May 2002 and December 2018, the immunosuppression regimens consisted of induction with an anti-CD25 antibody (basiliximab), followed by maintenance treatment with corticosteroid, calcineurin inhibitor and mycophenolate mofetil^47^.

### Prophylactic maneuver for recurrence of FSGS

In four of nine patients with primary FSGS, two to four sessions of plasmapheresis were performed prior to living-donor kidney transplantation. A single dose of rituximab (375 mg/m^2^) was also administered in one patient before living-donor kidney transplantation in 2012^50^. Patients with genetic FSGS did not receive the prophylactic maneuver.

### Whole-exome analysis

Whole-exome analysis was performed using a previously described method^11,42^. In brief, genomic DNA was extracted from peripheral blood. Exon capture was performed with a commercial kit (SureSelect Human All Exon Kit v5; Agilent Technologies, Santa Clara, CA, USA). Exon libraries were sequenced (HiSeq 2000 platform; Illumina, San Diego, CA, USA) according to the manufacturer’s instructions. Paired 100-base pair reads were aligned to the reference human genome (University of California Santa Cruz hg19) using the Burrows–Wheeler Aligner (Version 0.7.3a)^51^. Single-nucleotide variants and indels were identified as previously described^52^. We focused on the variants of 64 genes associated with FSGS and steroid-resistant nephrotic syndrome (Supplementary Table 3). Mitochondrial genome was not interrogated. Next, variant filtering on the basis of population frequency was performed to include only minor allele frequencies of < 1% of healthy control population databases^53,54^. Variants that were protein-truncating, highly conserved across species, and predicted to be deleterious based on at least two of three programs’ prediction scores from the web-based prediction programs PolyPhen-2 (http://genetics.bwh.harvard.edu/pph2), SIFT (Sorting Intolerant From Tolerant) (http://sift.bii.a-star.edu.sg/), and MutationTaster (http://www.mutationtaster.org) were kept for analysis.

### Degree of FPE in genetic FSGS literature review

We performed a comprehensive literature search of the PubMed database (up to June 2020) to identify review articles, original articles and case reports that described the degree of FPE in FSGS patients with identified mutated genes that were analyzed in the present study (Supplementary Table 3). Articles and reports that described patients with congenital or infantile nephrotic syndrome were excluded. We developed a search strategy that used a combination of text words and Medical Subject Headings, which included the following: “genetic,” “genetic testing,” “genes,” “focal segmental glomerulosclerosis,” and each name of 64 genes listed in Supplementary Table 3. The search was limited to human studies published in English. We further reviewed the reference lists of the selected studies for additional publications.

### Statistical analysis

Statistical analysis was performed for the comparisons between primary FSGS and genetic FSGS patients. Data were expressed as medians with 25th and 75th percentiles and were compared using the Mann–Whitney U test. Categorical data were analyzed using chi-squared test or Fisher’s exact test as appropriate. Spearman’s rank correlation coefficients were calculated to assess the relationship between the amount of proteinuria and the degree of FPE. For all statistical tests, a *p* value < 0.05 was considered statistically significant.

## Supplementary Information

Supplementary Information.

## Data Availability

The dataset generated and analyzed in the current study are available from the corresponding author upon reasonable request.
