# Supplementary material for: Degree of foot process effacement in patients with genetic focal segmental glomerulosclerosis: a single-center analysis and review of the literature
Source: Sci Rep. 2021 Jun 8;11:12008. doi: 10.1038/s41598-021-91520-9 (PMC8187362; doi:10.1038/s41598-021-91520-9)
Supplement: Supplementary file 1 — Supplementary Information. [file 41598_2021_91520_MOESM1_ESM.docx]

**Degree of foot process effacement in patients with genetic focal segmental glomerulosclerosis:**

**a single-center analysis and review of the literature**

**Supplementary Material**

Kiyonobu Ishizuka, M.D.^1^, Kenichiro Miura, M.D. Ph.D.^1^, Taeko Hashimoto, M.D. Ph.D.^2^, Naoto Kaneko, M.D.^1^, Yutaka Harita, M.D. Ph.D.^3^, Tomoo Yabuuchi, M.D.^1^, Masataka Hisano, M.D. Ph.D.^4^, Shuichiro Fujinaga, MD. Ph.D.^5^, Tae Omori, M.D.^6^, Yutaka Yamaguchi, M.D. Ph.D.^7^, and Motoshi Hattori, M.D. Ph.D.^1*^

1. Department of Pediatric Nephrology, Tokyo Women’s Medical University, School of Medicine, Tokyo, Japan
2. Department of Pediatrics, Yamagata University School of Medicine, Yamagata, Japan
3. Department of Pediatrics, Graduate School of Medicine, The University of Tokyo, Tokyo, Japan
4. Department of Nephrology, Chiba Children’s Hospital, Chiba, Japan
5. Division of Nephrology, Saitama Children’s Medical Center, 1-2 Shintoshin, Chuo-ku, Saitama city, Saitama, 330-8777, Japan
6. Department of Pediatrics, Tokyo Metropolitan Bokutoh Hospital, Tokyo, Japan
7. Yamaguchi’s Pathology Laboratory, Chiba, Japan

| Supplementary Table 1. Pathogenic mutations in FSGS/SRNS associated genes identified in this study | | | | | |  |
| --- | --- | --- | --- | --- | --- | --- |
| No. | Gene | Zygosity | c. Change**/** p. Change | PP2 SIFT/MutationTaster | Allele Frequency in gnomAD | References |
| 1 | *NUP107* | Cpd het | c.1079_1083delAAGAG  p.Glu360Glyfs*6  c.2492A>C  p.Asp831Ala | - -/DC  1 D/DC | 1/250606  3/251334 | Miyake et al. *Am. J. Hum. Genet*. 2015  Miyake et al. *Am. J. Hum. Genet*. 2015 |
| 2 | *NUP107* | Cpd het | c.1079_1083delAAGAG  p.Glu360Glyfs*6  c.2492A>C  p.Asp831Ala | - -/DC  1 D/DC | 1/250606  3/251334 | Miyake et al. *Am. J. Hum. Genet*. 2015  Miyake et al. *Am. J. Hum. Genet.* 2015 |
| 3 | *NUP107* | Cpd het | c.2030G>A  p.Trp677*  c.2492A>C  p.Asp831Ala | - -/DC  1 D/DC | N/A  3/251334 | novel  Miyake et al. *Am. J. Hum. Genet*. 2015 |
| 4 | *WT1* | Het | c.1392C>A  p.Asp464Glu | 0.969 D/DC | N/A | Lipska et al. *Kidney. Int*. 2014 |
| 5 | *WT1* | Het | c.1139G>A  p.Arg380Gln | 0.939 D/DC | N/A | novel |
| 6 | *LAMB2* | Cpd het | c.2095G>C  p.Gly699Arg  c.1406G>A  p.Arg469Gln | 0.986 T/DC  0.99 D/DC | 248/283848  N/A | Ogino et al. *J. Hum. Genet*. 2016  Ogino et al. *J. Hum. Genet*. 2016 |
| 7 | *INF2* | Het | c.550G>A  p.Glu184Lys | 1D/DC | N/A | Brown et al. *Nat. Genet*. 2010 |
| 8 | *NUP93* | Cpd het | c.1573C>T  p.Arg525Trp  c.1886A>G  p.Trp629Cys | 0.999 D/DC  1 D/DC | 2/251246  1/250624 | Hashimoto et al. *Kidney. Int. Rep*. 2019  Hashimoto et al. *Kidney. Int. Rep*. 2019 |

Cpd het, compound heterozygous; D, deleterious; DC, disease causing; gnomAD, The Gnome Aggregation Database; Het, heterozygous; N/A, not available; PP2, Polyphen-2; SIFT, Sorting Intolerant From Tolerant; T, tolerant

| Supplementary Table 2. Clinical characteristics of patients with primary FSGS included in this study | | | | | | | | | |
| --- | --- | --- | --- | --- | --- | --- | --- | --- | --- |
| Patient | 9 | 10 | 11 | 12 | 13 | 14 | 15 | 16 | 17 |
| Age at onset (yrs) | 11.2 | 3.0 | 7.3 | 8.8 | 6.7 | 4.1 | 3.4 | 4.2 | 1.3 |
| Sex | M | M | F | M | F | M | M | M | M |
| Time from onset to ESKD (yrs) | 1.6 | 3.0 | 1.9 | 7.9 | 6.5 | 1.1 | 10.9 | 11.8 | 7.7 |
| Serum TP level (g/dl) at kidney biopsy | 3.5 | 3.8 | 3.4 | 3.5 | 4.5 | 3.4 | 4.7 | 3.1 | 6.0 |
| Urinary protein to creatinine ratio (g/g) at kidney biopsy | 30.0 | 8.3 | 10.0 | 9.8 | 10.5 | 5 | 9.1 | 14.6 | 0.6 |
| Columbia classification of FSGS | NOS | COL | COL | COL | COL | NOS | COL | CEL | NOS |
| Edema during clinical course | Yes | Yes | Yes | Yes | Yes | Yes | Yes | Yes | Yes |
| Age at kidney transplantation (yrs) | 13.6 | 9.8 | 12.5 | 16.9 | 14.0 | 8.0 | 24.9 | 19.3 | 13.0 |
| Donor type | LD | DD | LD | LD | DD | DD | LD | LD | LD |
| Time from kidney transplantation to recurrence (days) | 0 | 0 | 0 | 0 | 4 | 9 | 20 | 1 | 0 |
| Maximum proteinuria (g/g Cr) at recurrence | 13.0 | 32.2 | 60.0 | 77.0 | 35.7 | 6.0 | 20.0 | 8.2 | 86.7 |

CEL, cellular variant; COL, collapsing variant; DD, deceased donor; ESKD, end-stage kidney disease; F, female; FSGS, focal segmental glomerulosclerosis;

LD, living donor; M, male; NOS, not otherwise specified; TIP, tip variant; TP, total protein; Cr, creatinine.

| Supplementary Table 3. List of 64 genes associated with FSGS/SRNS used for whole exome sequencing in this study | | | | |
| --- | --- | --- | --- | --- |
| **Gene** | **OMIM_ID** | **Mode of inheritance** | **Protein** | **Reference** |
| *ACTN4* | 604638 | AD | alpha-actinin 4 | [1] |
| *ADCK4* | 615567 | AR | aarF domain-containing kinase 4 | [2] |
| *ANKFY1* | 607927 | AR | ankyrin repeats- and FYVE domain-containing protein 1 | [3] |
| *ANLN* | 616027 | AD | actin-binding protein anillin | [4] |
| *ARHGAP24* | 610586 | AD | Rho GTPase-activating protein 24 | [5] |
| *ARHGDIA* | 601925 | AR | Rho GDP-dissociation inhibitor alpha | [6] |
| *AVIL* | 613397 | AR | advillin | [7] |
| *CD151* | 609057 | AR | CD151 antigen | [8] |
| *CD2AP* | 604241 | AD/AR | CD2-associated protein | [9] |
| *COL4A3* | 120070 | AD/AR | collagen, type iv, alpha-3 | [10] |
| *COL4A4* | 120131 | AD/AR | collagen, type iv, alpha-4 | [10] |
| *COL4A5* | 303630 | XLD | collagen, type iv, alpha-5 | [10] |
| *COQ2* | 609825 | AR | coenzyme q2, polyprenyltransferase | [11] |
| *COQ6* | 614647 | AR | coenzyme q6, monooxygenase | [12] |
| *CRB2* | 609720 | AR | crumbs homolog 2 | [13] |
| *CUBN* | 602997 | AR | cubilin | [14] |
| *DLC1* | 604258 | AR | deleted in liver cancer 1 | [15] |
| *EMP2* | 602334 | AR | epithelial membrane protein 2 | [16] |
| *FAT1* | 600976 | AR | fat atypical cadherin 1 | [17] |
| *GAPVD1* | 611714 | AR | GTPase-activating protein and vps9 domains 1 | [3] |
| *GON7* | 617436 | AR | c14orf142;chromosome 14 open reading frame 142 | [18] |
| *INF2* | 610982 | AD | inverted formin 2 | [19] |
| *ITGA3* | 605025 | AR | integrin, alpha-3 | [20] |
| *ITGB4* | 147557 | AR | integrin, beta-4 | [21] |
| *ITSN1* | 602442 | AR | intersectin 1 | [15] |
| *ITSN2* | 604464 | AR | intersectin 2 | [15] |
| *KANK1* | 607704 | AR | kn motif- and ankyrin repeat domain-containing protein 1 | [22] |
| *KANK2* | 614610 | AR | kn motif- and ankyrin repeat domain-containing protein 2 | [22] |
| *KANK4* | 614612 | AR | kn motif- and ankyrin repeat domain-containing protein 4 | [22] |
| *KIRREL1* | 607428 | AR | kin of IRRE-like protein 1 | [23] |
| *LAGE3* | 300060 | XLR | L antigen family, member 3 | [24] |
| *LAMA5* | 601033 | AR | laminin, alpha-5 | [25] |
| *LAMB2* | 150325 | AR | laminin, beta-2 | [26] |
| *LMX1B* | 602575 | AD | lim homeobox transcription factor 1, beta | [27] |
| *MAGI2* | 606382 | AR | membrane-associated guanylate kinase, WW, and PDZ domain-containing 2 | [15] |
| *MYO1E* | 601479 | AR | myosin 1E | [28] |
| *NPHS1* | 602716 | AR | nephrin | [29] |
| *NPHS2* | 604766 | AR | podocin | [30] |
| *NUP107* | 607617 | AR | nucleoporin, 107-kd | [31] |
| *NUP205* | 614352 | AR | nucleoporin, 205-kd | [32] |
| *NUP93* | 614351 | AR | nucleoporin, 93-kd | [32] |
| *NUP133* | 607613 | AR | nucleoporin, 133-kd | [33] |
| *NUP85* | 170285 | AR | nucleoporin, 85-kd | [33] |
| *NXF5* | 300319 | XLR | nuclear RNA export factor 5 | [34] |
| *OSGEP* | 610107 | AR | o-sialoglycoprotein endopeptidase | [24] |
| *PAX2* | 167409 | AD | paired box protein pax-2 | [35] |
| *PDSS2* | 610564 | AR | prenyl diphosphate synthase, subunit 2 | [36] |
| *PLCE1* | 608414 | AR | phospholipase c, epsilon-1 | [37] |
| *PODXL* | 602632 | AD | podocalyxin like protein | [38] |
| *PTPRO* | 600579 | AR | protein-tyrosine phosphatase, receptor-type, o | [39] |
| *SCARB2* | 602257 | AR | scavenger receptor class b, member 2 | [40] |
| *SGPL1* | 603729 | AR | sphingosine-1-phosphate lyase 1 | [41] |
| *SMARCAL1* | 606622 | AR | swi/snf-related, matrix-associated, actin-dependent regulator of chromatin, subfamily a-like protein 1 | [42] |
| *TNS2(TENC1)* | 607717 | AR | tensin 2 | [15] |
| *TP53RK* | 608679 | AR | tp53-regulating kinase | [24] |
| *TPRKB* | 608680 | AR | tp53rk-binding protein | [24] |
| *TRPC6* | 603652 | AD | transient receptor potential cation channel, subfamily c, member 6 | [43] |
| *TTC21B* | 612014 | AR | tetratricopeptide repeat domain-containing protein 21b | [44] |
| *WDR4* | 605924 | AR | wd repeat-containing protein 4 | [45] |
| *WDR73* | 616144 | AR | wd repeat-containing protein 73 | [46] |
| *WT1* | 607102 | AD | Wilms’ tumour protein 1 | [47] |
| *XPO5* | 607845 | AR | exportin 5 | [32] |
| *YRDC* | 612276 | AR | YrdC domain-containing protein | [18] |
| *ZMPSTE24* | 606480 | AR | zinc metalloproteinase ste24 | [48] |

AD, autosomal dominant; AR, autosomal recessive; FSGS, focal segmental glomerulosclerosis; SRNS, steroid resistant nephrotic syndrome; XLD, X-linked dominant; XLR, X-linked recessive

Reference articles of each gene

[1] Kaplan, J.M. *et al*. Mutations in *ACTN4*, encoding alpha-actinin-4, cause familial focal segmental glomerulosclerosis. *Nat. Genet*. **24**, 251-256 (2000).

[2] Ashraf, S. *et al*. *ADCK4* mutations promote steroid-resistant nephrotic syndrome through CoQ10 biosynthesis disruption. *Clin. Invest*. **23**, 5179-5189 (2013).

[3] Hermle, T. *et al*. *GAPVD1* and *ANKFY1* mutations implicate RAB5 regulation in nephrotic syndrome. *J. Am. Soc. Nephrol*. **29**, 2123-2138 (2018).

[4] Gbadegesin, R.A. *et al*. Mutations in the gene that encodes the F-actin binding protein anillin cause FSGS. *J. Am. Soc. Nephrol*. **25**, 1991-2002 (2014).

[5] Akilesh, S. *et al*. Arhgap24 inactivates Rac1 in mouse podocytes, and a mutant form is associated with familial focal segmental glomerulosclerosis. *J. Clin. Invest*. **121**, 4127-4137 (2011).

[6] Gee, H.Y. *et al*. *ARHGDIA* mutations cause nephrotic syndrome via defective RHO GTPase signaling. *J. Clin. Invest*. **123**, 3243-3253 (2013).

[7] Rao, J. *et al*. Adillin acts upstream of phospholipase C ε1 in steroid-resistant nephrotic syndrome. *J. Clin. Invest*. **127**, 4257-4269 (2017).

[8] Crew, V.K. *et al*. CD151, the first member of the tetrapanin (TM4) superfamily detected on erythrocytes, is essential for the correct assembly of human basement membranes in kidney and skin. *Blood*. **104**, 2217-2223 (2004).

[9] Kim, J.M. *et al*. CD2-associated protein haploinsufficiency is linked to glomerular disease susceptibility. *Science*. **300**, 1298-1300 (2003).

[10] Gast, C. *et al*. Collagen (*COL4A*) mutations are the most frequent mutations underlying adult focal segmental glomerulosclerosis. *Nephrol. Dial. Transplant*. **31**, 961-970 (2016).

[11] Diomedi-Camassei, F. *et al*. COQ2 nephropathy: a newly described inherited mitochondriopathy with primary renal involvement. *J. Am. Soc. Nephrol*. **18**, 2773-2780 (2007).

[12] Heeringa, S.F. *et al*. *COQ6* mutations in human patients produce nephrotic syndrome with sensorineural deafness. *J. Clin. Invest*. **121**, 2013-2024 (2011).

[13] Ebarasi, L. *et al*. Defects of *CRB2* cause steroid-resistant nephrotic syndrome. *Am. J. Hum. Genet*. **96**, 153-161 (2015).

[14] Bedin, M. *et al*. Human C-terminal *CUBN* variants associate with chronic proteinuria and normal renal function. *J. Clin. Invest*. **130**, 335-344 (2020).

[15] Ashraf, S. *et al*. Mutations in six nephrosis genes delineate a pathogenic pathway amenable to treatment. *Nat. Commun*. **9,** 1960 (2018).

[16] Gee, H.Y. *et al*. Mutations in *EMP2* cause childhood-onset nephrotic syndrome. *Am. J. Hum. Genet*. **94**: 884-890 (2014).

[17] Gee, H.Y. *et al*. *FAT1* mutations cause a glomerulotubular nephropathy. *Nat. Commun*. **7**, 10822 (2016).

[18] Arrondel, C. *et al*. Defects in t^6^ A tRNA modification due to *GON7* and *YRDC* mutations lead to Galloway-Mowat syndrome. *Nat. Commun*. **10**, 3967 (2019).

[19] Brown, E.J. *et al*. Mutations in the formin gene *INF2* cause focal segmental glomerulosclerosis. *Nat. Genet*. **42**, 72-76 (2010).

[20] Yalcin, E.G. *et al*. Crucial role of posttranslational modifications of integrin α3 in interstitial lung disease and nephrotic syndrome. *Hum. Mol. Genet*. **24**, 3679-3688 (2015).

[21] Kambham, N. *et al*. Congenital focal segmental glomerulosclerosis associated with beta4 integrin mutation and epidermolysis bullosa. *Am. J. Kidney. Dis*. **36**, 190-196 (2000).

[22] Gee, H.Y. *et al*. *KANK* deficiency leads to podocyte dysfunction and nephrotic syndrome. *J. Clin. Invest*. **125**, 2375-2384 (2015).

[23] Solanki, A.K. *et al*. Mutations in *KIRREL1*, a slit diaphragm component, cause steroid-resistant nephrotic syndrome. *Kidney. Int*. **96**, 883-889 (2019).

[24] Braun, D.A. *et al*. Mutations in *KEOPS*-complex genes cause nephrotic syndrome with primary microcephaly. *Nat. Genet*. **49**, 1529-1538 (2017).

[25] Braun, D.A. *et al*. Genetic variants in the *LAMA5* gene in pediatric nephrotic syndrome. *Nephrol. Dial. Transplant*. **34**, 485–493 (2019).

[26] Zenker, M. *et al*. Human *laminin beta2* deficiency causes congenital nephrosis with mesangial sclerosis and distinct eye abnormalities. *Hum. Mol. Genet*. **13**, 2625-2632 (2004).

[27] Boyer, O. *et al*. *LMX1B* mutations cause hereditary FSGS without extrarenal involvement. *J. Am. Soc. Nephrol*. **24**, 1216-1222 (2013).

[28] Mele, C. *et al*. *MYO1E* mutations and childhood familial focal segmental glomerulosclerosis. *N. Engl. J. Med*. **365**, 295-306 (2011).

[29] Santίn, S. *et al*. Nephrin mutations cause childhood- and adult- onset focal segmental glomerulosclerosis. *Kidney. Int*. **76**, 1268-1276 (2009).

[30] Boute, N. *et al*. *NPHS2*, encoding the glomerular protein podocin, is mutated in autosomal recessive steroid-resistant nephrotic syndrome. *Nat. Genet*. **24**, 349-354 (2009).

[31] Miyake, N. *et al*. Biallelic mutations in nuclear pore complex subunit *NUP107* cause early-childhood-onset steroid-resistant nephrotic syndrome. *Am. J. Hum. Genet*. **97**, 555-566 (2015).

[32] Braun, D.A. *et al*. Mutations in nuclear pore genes *NUP93*, *NUP205* and *XPO5* cause steroid-resistant nephrotic syndrome. *Nat. Genet*. **48**, 457-465 (2016).

[33] Braun, D.A. *et al*. Mutations in multiple components of the nuclear pore complex cause nephrotic syndrome. *J. Clin. Invest*. **128**, 4313-4328 (2018).

[34] Esposito, T. *et al*. Unique X-linked familial FSGS with co-segregating heart block disorder is associated with a mutation in the NXF5 gene. *Hum. Mol. Genet*. **22**, 3654-3666 (2013).

[35] Barua, M, *et al*. Mutations in *PAX2* associated with adult-onset FSGS. *J. Am. Soc. Nephrol*. **25**, 1942-1953 (2014).

[36] Lványi, B. *et al*. Diffuse mesangial sclerosis in a *PDSS2* mutation-induced coenzyme Q10 deficiency. *Pediatr. Nephrol*. **33**, 439-446 (2018).

[37] Hinkes, B. *et al*. Positional cloning uncovers mutations in *PLCE1* responsible for a nephrotic syndrome variant that may be reversible. *Nat. Genet*. **38**, 1397-1405 (2006).

[38] Lin, F.J. *et al*. First identification of *PODXL* nonsense mutations in autosomal dominant focal segmental glomerulosclerosis. *Clin. Sci. (Lond)*. **133**, 9-21 (2019).

[39] Ozaltin, F. *et al*. Disruption of *PTPRO* causes childhood-onset nephrotic syndrome. *Am. J. Hum. Genet*. **89,** 139-147 (2011).

[40] Berkovic, S.F. *et al*. Array-based gene discovery with three unrelated subjects shows *SCARB2*/*LIMP-2* deficiency causes myoclonus epilepsy and glomerulosclerosis. *Am. J. Hum. Genet*. **82**, 673-684 (2008).

[41] Lovric, S. *et al*. Mutations in sphingosine-1-phosphate lyase cause nephrosis with ichthyosis and adrenal insufficiency. *J. Clin. Invest*. **127**, 912-928 (2017).

[42] Lipska-Ziętkiewicz, B.S. *et al*. Low renal but high extrarenal phenotype variability in Schimke immuno-osseous dysplasia. *PLoS. One*. **12**, e0180926 (2017).

[43] Winn, M.P. *et al*. A mutation in the *TRPC6* cation channel causes familial focal segmental glomerulosclerosis. *Science*. **308**, 1801-1804 (2005).

[44] Cong, E.H. *et al*. A homozygous misssense mutation in the ciliary gene *TTC21B* causes familial FSGS. *J. Am. Soc. Nephrol*. **25**, 2435-2443 (2014).

[45] Braun, D.A. *et al*. Mutations in *WDR4* as a new cause of Galloway-Mowat syndrome. *Am. J. Med. Genet. A*. **176**, 2460-2465 (2018).

[46] Colin, E. *et al*. Loss-of-function mutations in *WDR73* are responsible for microcephaly and steroid-resistant nephrotic syndrome: Galloway-Mowat syndrome. *Am. J. Hum. Genet*. **95**, 637-648 (2014).

[47] Lipska, B.S. *et al*. Genotype-phenotype associations in WT1 glomerulopathy. *Kidney. Int*. **85**, 1169-1178 (2014).

[48] Agarwal, A.K. *et al*. Focal segmental glomerulosclerosis in patients with mandibuloacral dysplasia owing to *ZMPSTE24* deficiency. *J. Investig. Med*. **54**, 208-213 (2006).

| Supplementary Table 4. Clinical characteristics of patients with genetic FSGS included in this study | | | | | | | | |
| --- | --- | --- | --- | --- | --- | --- | --- | --- |
| Patient | 1 | 2 | 3 | 4 | 5 | 6 | 7 | 8 |
| Age at onset (yrs) | 3.1 | 3.0 | 2.8 | 3.5 | 7.0 | 1.8 | 10.8 | 4.9 |
| Sex | F | M | F | M | F | M | M | F |
| Time from onset to ESKD (yrs) | 2.2 | 2.1 | 8.6 | 7.9 | 5.8 | 0.2 | 6.1 | 1.2 |
| Serum TP level (g/dl) at kidney biopsy | 4.5 | 4.9 | 6.4 | 6.1 | 5.6 | 3.9 | 6.7 | 5.3 |
| Urinary protein to creatinine ratio (g/g) at kidney biopsy | 7.0 | 16.4 | 1.3 | 4.0 | 5.9 | 2.5 | 1.8 | 2.5 |
| Columbia classification of FSGS | COL | COL | NOS | PH | PH | NOS | PH | COL |
| Edema during clinical course | Yes | No | No | No | No | Yes | No | No |
| Age at kidney transplantation (yrs) | 9.5 | 8.7 | 14.6 | 13.1 | 15.8 | 7.3 | 20.2 | 15.1 |
| Donor type | LD | LD | DD | LD | DD | LD | LD | LD |

COL, collapsing variant; DD, deceased donor; ESKD, end-stage kidney disease; F, female; FSGS, focal segmental glomerulosclerosis;

LD, living donor; M, male; NOS, not otherwise specified; PH, perihilar; TIP, tip variant; TP, total protein; Cr, creatinine.

Supplementary Fig.1


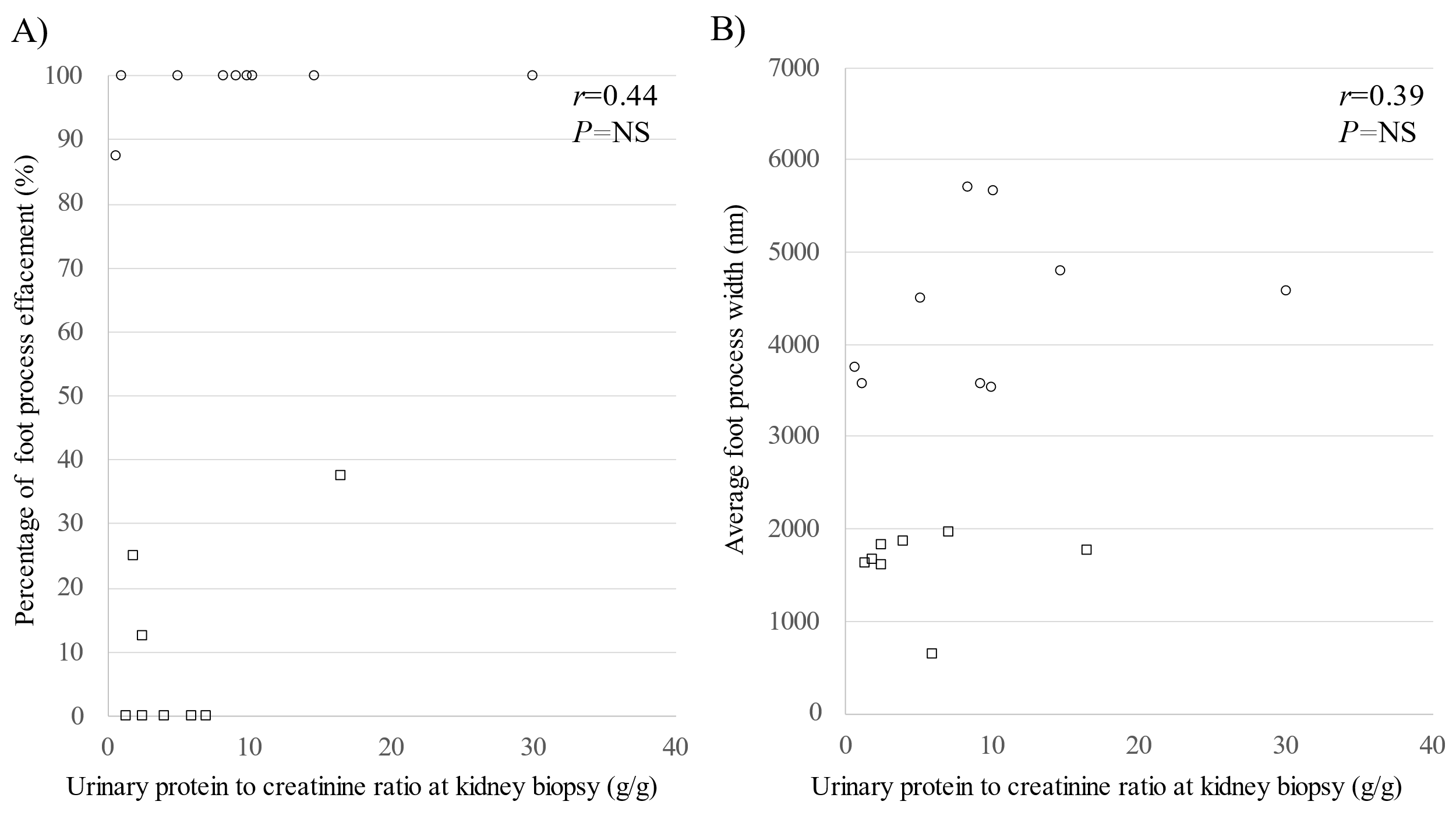


**Supplementary Fig. 1 The relationship between the amount of proteinuria and the degree of foot process effacement**

The amount of proteinuria correlated with neither percentage of foot process effacement (*r*=0.44; *p*=NS) (A) nor average foot process width (*r*=0.39; *p*=NS) (B). Circles and squares denote patients with primary FSGS and genetic FSGS, respectively
